# The Epigenetic Control of Hepatitis B Virus Modulates the Outcome of Infection

**DOI:** 10.3389/fmicb.2015.01491

**Published:** 2016-01-06

**Authors:** Lemonica Koumbi, Peter Karayiannis

**Affiliations:** ^1^Hepatology and Gastroenterology Section, Department of Medicine, Imperial CollegeLondon, UK; ^2^Microbiology/Molecular Virology, University of Nicosia Medical SchoolNicosia, Cyprus

**Keywords:** hepatitis B virus, epigenetics, methylation, cccDNA, histone, acetylation

## Abstract

Epigenetic modifications are stable alterations in gene expression that do not involve mutations of the genetic sequence itself. It has become increasingly clear that epigenetic factors contribute to the outcome of chronic hepatitis B virus (HBV) infection by affecting cellular and virion gene expression, viral replication and the development of hepatocellular carcinoma. HBV persists in the nucleus of infected hepatocytes as a stable non-integrated covalently closed circular DNA (cccDNA) which functions as a minichromosome. There are two major forms of HBV epigenetic regulation: posttranslational modification of histone proteins associated with the cccDNA minichromosome and DNA methylation of viral and host genomes. This review explores how HBV can interphase with host epigenetic regulation in order to evade host defences and to promote its own survival and persistence. We focus on the effect of cccDNA bound-histone modifications and the methylation status of HBV DNA in regulating viral replication. Investigation of HBV epigenetic control has important clinical correlates with regards to the development of potential therapeutic regimens that will successfully eradicate HBV infection and deal with HBV reactivation in those undergoing treatment with demethylating agents.

## Introduction

Hepatitis B virus (HBV) is a highly transmissible pathogen infecting humans for more than 1500 years (Zhou and Holmes, 2007). Despite the availability of a prophylactic vaccine for more than three decades now, HBV continues to pose one of the most prevalent health problems with about 240 million people worldwide chronically infected and accounting for over 600,000 deaths per year (WHO, 2015). Current therapeutic regimens include pegylated-IFN-α (PegIFN-α) and nucleos(t)ide analogs (NAs). Both types of antiviral treatment are not capable of eliminating the virus and do not establish long-term control of infection after treatment withdrawal in the majority of patients.

HBV is the prototype of the *hepadnaviridae* family and has evolved a distinctive and successful replication strategy, which allows its indefinite persistence in the liver of the infected host. Upon infection of the hepatocyte, the HBV genome translocates to the nucleus, where its relaxed circular, partially double stranded DNA is converted into a covalently closed circular DNA (cccDNA) molecule. The cccDNA is the template for the synthesis of six co-terminal mRNA transcripts (Tuttleman et al., 1986; Seeger and Mason, 2000). One of the transcripts, termed pre-genomic RNA (pgRNA), is the template for genome replication and encodes for the core and polymerase proteins. Translation of the transcripts occurs in the cytoplasm and the encapsidation of the pgRNA into core particles follows (Levrero et al., 2009). Inside the core particle, the viral polymerase directs the synthesis of the minus DNA strand of the genome by reverse transcription of the pgRNA template, which then serves as the template for plus DNA strand synthesis. Mature core particles containing DNA genomes are then enveloped and released or cycled back to the nucleus to replenish the cccDNA pool (Tuttleman et al., 1986). Persistent infection of HBV relies on the stable maintenance and proper function of the cccDNA pool in the nucleus of the infected hepatocytes. Variable levels of cccDNA can be found in different phases of the natural history of chronic HBV patients. In HBeAg-negative patients or inactive carriers, cccDNA transcription is about 10-fold lower than that in HBeAg-positive patients, while cccDNA levels can be detected in cases with absent or very low viral replication (Werle-Lapostolle et al., 2004; Liu et al., 2013).

Amongst the smallest of all known virus genomes, the 3.2 kb HBV genome contains four partially overlapping open reading frames (ORFs). HBV transcription is regulated by its four promoters and by *cis*-acting viral elements including two enhancers (enhancer I and enhancer II) and a negative regulatory region that depend on host transcription factors for their function (Moolla et al., 2002). Additionally, a number of epigenetic modifications have been identified which regulate viral replication and viral gene expression. Non-integrated nuclear HBV DNA associates with histones combined with HBcAg to form stable cccDNA minichromosomes (Bock et al., 2001). Chromatin condensation of cccDNA is a critical step for the regulation of viral gene expression because it determines the accessibility of DNA to the regulatory transcription factors. The acetylation status of cccDNA bound histones controls HBV replication in a fashion identical to that seen in human genes; hypoacetylation correlates with low viral replication and hyperacetylation leads to increased HBV replication (Pollicino et al., 2006). In addition, both viral and host DNA are known to be targets for methylation in chronic HBV infection suggesting dual effects of methylation as potentially both protective and harmful for the host (Guo et al., 2009; Vivekanandan et al., 2009; Kaur et al., 2010). This review will provide an overview of how epigenetic factors, including genomic DNA methylation and histone modifications, contribute in HBV persistence and HBV-induced cancer as well as their possible therapeutic implications in chronic HBV infection.

## Chromatin Organization and Epigenetic Modifications

A number of epigenetic modifications have been recently identified that control viral replication in chronic HBV infection. Chromatin condensation of cccDNA is a critical step in the regulation of viral gene expression because it determines the accessibility of DNA to the regulatory transcription factors. It can be modulated through a variety of mechanisms, including posttranslational covalent modifications of histone tails, ATP-dependent chromatin remodeling events and recruitment of repressor factors on methylated DNA. Methylation is another common cellular defence mechanism in mammalian cells known to silence invading foreign DNA and viral genomes. It permits binding of protein complexes with chromatin-modulating properties and strictly depends on where the methyl group is located. Many host and viral promoters are enriched for CpG dinucleotides and methylation of cytidine leads to gene inactivation. Generally, DNA methylation at the promoter region leads to repression of gene expression, because the 5-methyl-cytosine interferes with the recognition and binding of transcriptional factors, to diminish mRNA transcription (Bird, 2002).

## Histone Acetylation

Histone modifications are all reversible and mainly localized at the amino-terminal histone tails. They include acetylation, methylation, phosphorylation, sumoylation, ubiquitination, ADP-ribosylation, deamination, and the non-covalent proline isomerization (histone H3) (Liu et al., 2012). To further increase the complexity, many of these modifications occur multiple times at the same residue of a histone tail and they influence gene expression patterns by two different mechanisms: (1) histone acetylation, which alters chromatin packaging allowing access to transcriptional machinery; and (2) by histone methylation, which generates interactions with chromatin-associated proteins.

Several enzymes catalyze these processes, including histone acetyltransferases (HATs), histone deacetylases (HDACs), histone methylatransfecrases (HMTs), and histone demethylases (HDMTs). HATs and HDACs regulate transcription by selectively acetylating or deacetylating the 𝜀-amino group of lysine residues in histone tails. Histone acetylation induced by HATs promotes chromatin opening and associates with gene transcription while histone hypoacetylation induced by HDACs diminishes the accessibility of the nucleosomal DNA to transcription factors and is associated with gene silencing (Berger et al., 2009).

## DNA Methylation

DNA methylation occurs at the 5-methyl cytosine predominantly in the context of CpG dinucleotides with S-adenosyl methionine as the methyl donor (Bird, 2002). CpG dinucleotides are often found accumulated in conserved regulatory regions (CpG islands) demonstrating their functional importance. The mammalian DNA methylation machinery consists of the DNA methyltransferases (DNMTs), which are responsible for the enzymatic addition of methyl groups, and the methyl-CpG-binding proteins (MBPs), which identify the methylation pattern (Egger et al., 2004). The DNMT family includes DNMT1, DNMT2, DNMT3A, DNMT3B, and DNMT3A. DNMT1 maintains the methylation pattern during cell division and methylates hemimethylated CpG islands. DNMT3A and DNMT3B can methylate unmethylated and hemimethylated CpG islands while DNMT2 lacks methyltransferase capabilities but plays a role in methylation of structural RNA (Jurkowska and Jeltsch, 2010).

DNA hypomethylation signifies one of the major DNA methylation states and refers to a relative situation that indicates a decrease from the “normal” methylation level. Hypomethylation in transcriptional regulatory regions generally induces gene silencing, either directly, by blocking the binding of transcription factors to their recognition sequences or indirectly, by preventing transcription factors from accessing their target sites through the attachment of MBPs. In turn, methyl-CpG binding domain proteins can recruit histone-modifying and chromatin remodeling complexes, such as HMTs and HDACs, to methylated sites resulting in histone methylation of certain amino acids in a histone (Lopez-Serra and Esteller, 2008). HMTs catalyze the transfer of methyl groups to histones and thus they regulate DNA methylation through chromatin-dependent transcription control. Histone methylation can cause transcription repression or activation depending on the target sites and thus serves in both epigenetic gene activation and silencing.

## Acetylation of the cccDna Minichromosome

**FIGURE 1:**
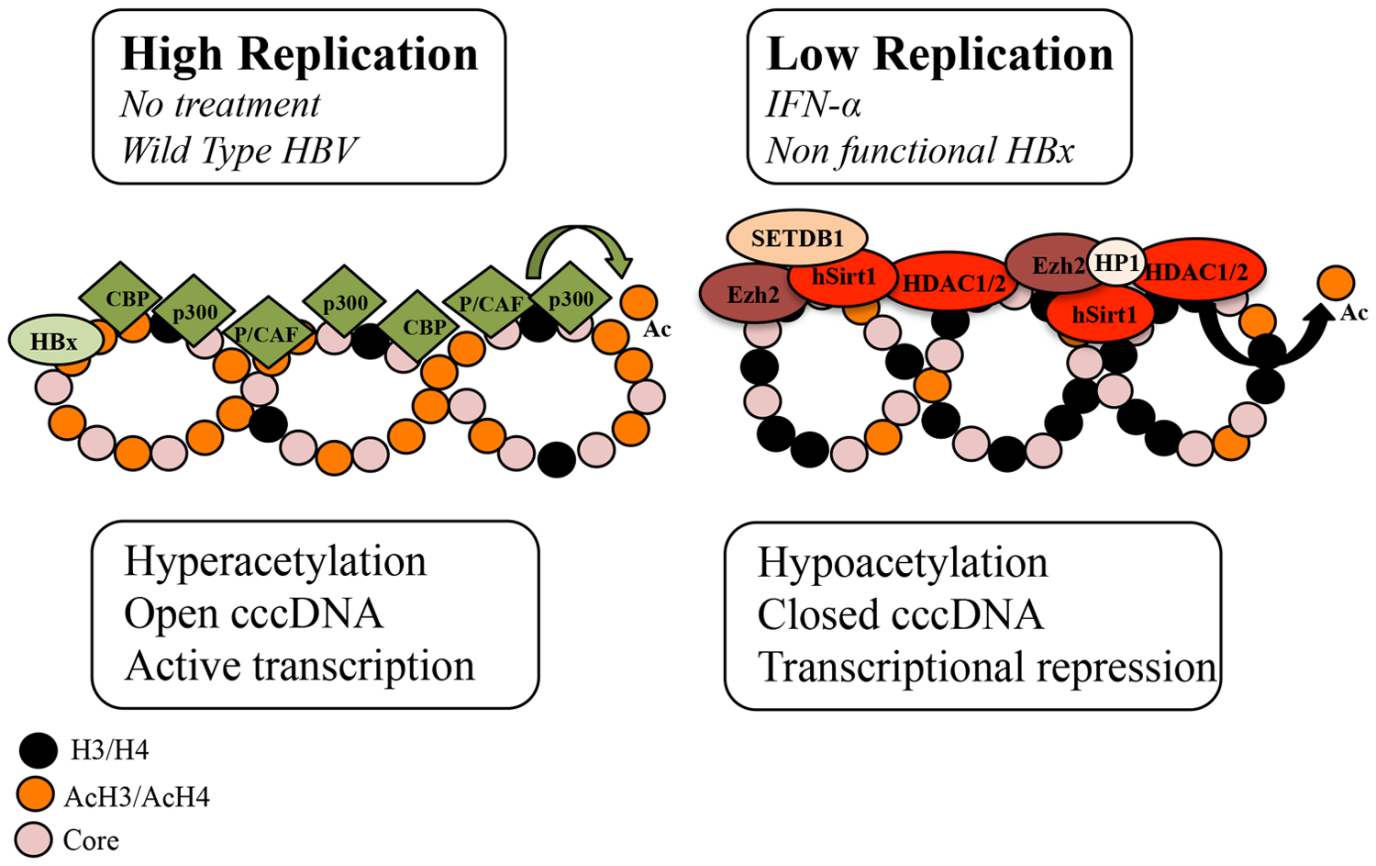
**Schematic representation of the chromatin changes on cccDNA in relation to viral replication.** The acetylation status of cccDNA-bound histones and the recruitment of chromatin modifying enzymes onto cccDNA change in response to IFNα treatment and HBx status. In high viral replication of the wild type virus or in the absence of IFNα treatment, cccDNA-bound histones are hyperacetylated, cccDNA-associated chromatin is in an open configuration and pgRNA is actively transcribed. In cells replicating an HBx mutant and in IFNα-treated cells, cccDNA-bound histones are hypoacetylated, the recruitment of the p300 acetyltransferase is severely impaired, whereas the recruitment of the histone deacetylases (HDACs) hSirt1 and HDAC1 as well as the polycomb protein enhancer of zeste homolog 2 (Ezh2) is increased. In the absence of HBx, hypoacetylation is accompanied by the recruitment of heterochromatin protein 1 factors (HP1) and SET domain, bifurcated 1(SETDB1). Modified from Belloni et al. (2009).

Hepatitis B virus cccDNA is formed by histone and non-histone proteins. Hypoacetylation of the cccDNA-associated H3 and H4 histones and the recruitment of the cellular acetyltransferases p300/CREB-binding protein (CBP) and HDAC1 onto cccDNA have been associated with low HBV replication both *in vivo* and *in vitro* (Pollicino et al., 2006). In addition, the presence of class I/II histone deacetylase inhibitors (valproic acid and trichostatin (TSA)) has been shown to induce an increase in HBV replication and the upregulation of cccDNA-bound H4 histones in a HepG2 cell based model (Pollicino et al., 2006) (**Figure 1**; **Table 1**). However, another study that used duck hepatitis B virus (DHBV) *in vitro* showed that a number of HDAC inhibitors, including TSA, suppressed cccDNA transcription and reduced HBV replication in a dose-response manner (Yan et al., 2012; Liu et al., 2013). Therefore it can be assumed that a cellular function sensitive to HDAC inhibitors is required for HBV RNA transcription.

**Table 1:** An outline of the main research findings on the (A) methylation and (B) acetylation mechanisms involved in HBV infection.

	Reference
**(A) Findings on HBV DNA methylation**	
Methylation of integrated HBV DNA.	Chen et al., 1988
HBx induces DNMT activity and hypermethylation of tumor suppressor gene promoters via DNMT3A1 and DNMT3A2 methylation.	Park et al., 2007
Island II methylation correlates with low or no HBsAg production. cccDNA methylation correlates with viral gene expression levels.	Vivekanandan et al., 2008a
Methylation of integrated and non-integrated liver HBV DNA in islands I (60%) and II (50%). Unmethylated serum HBV DNA.	Vivekanandan et al., 2008b
cccDNA methylation correlates with HBeAg-positivity in CHB patients and impairs virion productivity.	Guo et al., 2009
HBV DNA is unmethylated in early carcinogenesis and highly methylated in cancer. Methylation of HBcAg and HBsAg genes inhibits their expression.	Fernandez et al., 2009
HBx recruits DNMT3A and induces the methylation and transcriptional silencing of IL-4 receptor and metallothionein-1F.	Zheng et al., 2009
HBx expression correlates with DNMT1 and DNMT3A and hypermethylation of the p16^INK4A^ promoter in CHB patients.	Zhu et al., 2010
cccDNA methylation is associated with HBV viremia and aging in cirrhotic CHB patients.	Kim et al., 2011
HBV DNA methylation correlates with decreased viral replication and gene expression.	Vivekanandan et al., 2009
Increased expression of DNMT3 down-regulates viral protein and pgRNA production. HBV induces DNMT overexpression and correlates with methylation of host CpG islands.	Vivekanandan et al., 2010
HBV DNA methylation in CHB implicates island I in14%, island II 0.6% and island III 3.7% of cases. HBV DNA is unmethylated in CHB and highly methylated in HBV-related cancer.	Kaur et al., 2010
HBV DNA is unmethylated in occult HBV. CpG island I methylation correlates with HCC development.	
HBV CpG island distribution differs between HBV genotypes.	Zhang et al., 2013
HBx induces the hypermethylation of the uPA promoter (via the recruitment of DNMT3A2) leading to liver regeneration impairment.	Park et al., 2013
**(B) Findings on the acetylation of the cccDNA minichromosome**	
Low HBV replication correlates with cccDNA hypoacetylation and the recruitment of p300/CBP and HDAC1. Histone deacetylase inhibitors restore HBV replication.	Pollicino et al., 2006
HBx recruitment onto cccDNA correlates with HBV replication and acetyltransferase upregulation. In the absence of HBx, HBV decreased replication correlates with cccDNA hypoacetylation; p300 inhibition; reduced pgRNA, and deacetylase increase.	Belloni et al., 2009
IFN-α treatment reduces DHBV acetylation of cccDNA-bound H3K9 and H3K27 histones but has no effect on cccDNA-bound H3K9me3 and H3K27me2 demethylases. HDAC inhibitors block DHBV cccDNA transcription but not the long-lasting IFN-α-induced suppression of cccDNA.	Liu et al., 2013
IFN-α inhibits viral transcription by cccDNA hypoacetylation through active recruitment onto cccDNA of HDAC and of the transcriptional repressor complex.	Belloni et al., 2012
IL6 induces cccDNA hypoacetylation and silencing by reducing the binding of transcription factors (HNF1α, HNF4α, and STAT3) onto cccDNA.	Palumbo et al., 2015
HBx recruitment onto cccDNA activates HBV transcription by counteracting chromatin-mediated transcriptional repression established by SETDB1, HP1 and H3K9me3.	Riviere et al., 2015
IFN-α represses HBV by reducing active PTMs in cccDNA and that this effect can be recapitulated with the C646 agent (inhibits p300/CBP) The repressive mark H3K27me3 is underrepresented in cccDNA	Tropberger et al., 2015

*DNMT, DNA methyltransferase; uPA, urokinase-type plasminogen activator; CBP, CREB-binding protein; pgRNA, pregenomic RNA; cccDNA, covalently closed circular DNA; DHBV, duck hepatitis B virus; HDAC, histone deacetylase; HNF1α, hepatocyte nuclear factor 1α; HNF4α, hepatocyte nuclear factor 4α; SETDB1, SET domain, bifurcated 1; HP1, heterochromatin protein 1 factors; and PTM, posttranslational covalent modifications.*

HBV replication is known to be strongly inhibited by the administration of IFN-α, a type I IFN, that engages the IFN-α/β receptor complex to modulate the transcription of the IFN-stimulating genes (ISGs) via the Jak/Stat signaling pathway. Recently it has been shown in HBV-replicating cells and in HBV-infected chimeric uPA/SCID mice that IFN-α inhibits cccDNA-driven transcription by targeting the epigenetic control of cccDNA with the involvement of the chromatin remodeling Polycomb Repressive Complex 2 (Belloni et al., 2012). In response to IFN-α, cccDNA-bound histones become hypoacetylated and both components of the transcriptional repressor complex, YY1 and Ezh2, and the HDAC1 and hSirt are actively recruited onto cccDNA (Belloni et al., 2012). However, another study, using a DHBV cell based model demonstrated that IFN-α reduced the acetylation of cccDNA-bound H3K9 and H3K27 histone residues but failed to induce cccDNA-bound H3K9me3 and H3K27me2 demethylases (Liu et al., 2013). In addition, HDAC inhibitors blocked DHBV cccDNA transcription but did not affect the long-lasting IFNα-induced suppression of cccDNA transcription (Liu et al., 2013). It is possible that the reduced acetylation of H3K9 and H3K27 by IFN-α is catalyzed by HDACs that are not susceptible to the HDAC inhibitors used in the DHBV study or that this reduction is due to the disruption of a dynamic acetylation and deacetylation of histone H3 through preventing the recruitment of HATs onto DHBV cccDNA (**Figure 1**) (Liu et al., 2013). Interestingly, a recent study demonstrated that IFN-α represses HBV by reducing active PTMs on the cccDNA minichromosome and that this effect can be recapitulated with treatment with a small epigenetic agent, C646, which specifically inhibits p300/CBP HATs (Tropberger et al., 2015). Nevertheless, these findings suggest that IFN-α induces a persistent condition of “active epigenetic control” of cccDNA, involving all HBV transcripts that may contribute to the persistent, yet reversible, “off-therapy” inhibition of HBV replication. Contrary to IFN-α, treatment with IL6, another activator of the intracellular Jak/Stat signaling pathway, reduces cccDNA acetylation and transcription but without affecting cccDNA chromatinization (Palumbo et al., 2015). Instead IL6 has been shown to inhibit cccDNA transcription by reducing the binding of essential transcription factors HNF1α and HNF4α to cccDNA and by redistributing STAT3 binding from the cccDNA to IL6 cellular target genes (Palumbo et al., 2015).

HBx, a pleiotropic regulatory protein, acts as a promiscuous transactivator of viral and cellular promoters and is found in the cytoplasm and the nucleus of infected hepatocytes (Cougot et al., 2007). HBx activates the transcription of host genes by interacting directly with nuclear transcription factors or by activating various signal transduction pathways in the cytoplasm. In addition to its *trans-* and *cis*-activating roles, HBx protein has been proven to be a potent epigenetic modifying factor in liver tissue. It has been reported to modify chromatin dynamics *in vivo* by favoring the transcription of a number of CREB-regulated genes via the recruitment of the cellular acetyltransferases CBP and p300 to their promoters (Cougot et al., 2007). HBx is reported to activate HBV transcription by its recruitment onto cccDNA, through recruitment of PCAF/GCN5, p300 and CBP acetyltransferases onto cccDNA and through the inhibition of cellular factors involved in chromatin regulation, such as PP1/HDAC1 complex (Belloni et al., 2009; Riviere et al., 2015). On the contrary, in the absence of HBx, HBV replication was suppressed and this decrease correlated with the rapid hypoacetylation of cccDNA-bound histones, the severe impairment of the p300 recruitment and the reduced transcription of pgRNA from cccDNA, whereas the recruitment of the HDACs hSirt1 and HDAC1 preceded (Belloni et al., 2009). Notably, the IFN-α-induced cccDNA repression through hSirt and HDAC1 up-regulation was reported to be HBx independent (Belloni et al., 2012). A recent study showed that in the absence of HBx, HBV silencing associated not only with the deacetylation of histones but also with deposition of repressive chromatin markers (H3K9me2 and H3K9me3), the recruitment of heterochromatin protein 1 factors and the recruitment of SET domain, bifurcated 1 (SETDB1) histone methylatransferases that methylate H3K9 histone (Riviere et al., 2015). Interestingly, SETDB1 has been shown to be an oncogene and is significantly associated with HCC disease progression (Wong et al., 2015).

## HBV DNA Methylation

In chronic HBV infection, viral DNA methylation has been identified as a novel host defence mechanism, leading to the downregulation of viral gene expression. However, the association of HBV DNA methylation with the methylation of host genes and the development of cancer imply a harmful effect on the host. HBV DNA can be methylated in human tissues in both non-integrated forms (Vivekanandan et al., 2008b) and following integration into the human tissue (Chen et al., 1988).

## Non-Integrated HBV Genome

**FIGURE 2:**
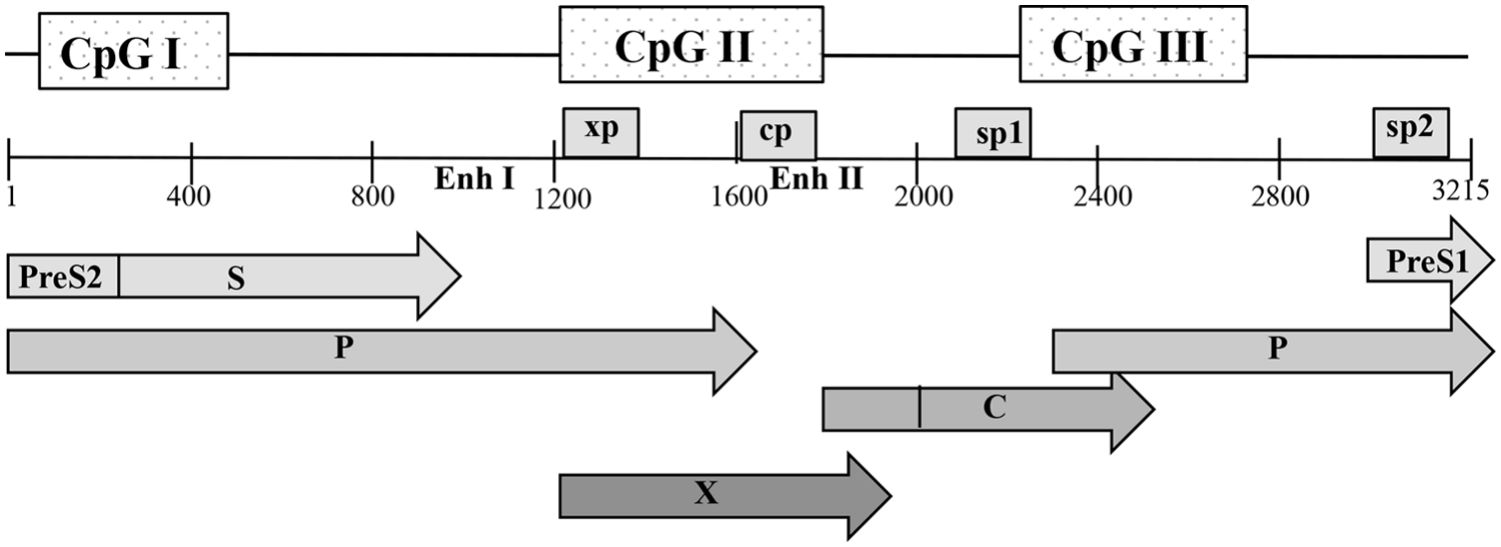
**Schematic representation of the open reading frames (ORFs) of pre-core/core, polymerase (P), surface (S), and X proteins with the genome in open configuration; the four promoters, cp, sp1, sp2, and xp, as well as the main regulatory elements, enhancers I and II (Enh I and Enh II), are indicated.** The positions of the three predicted CpG islands within the HBV genome are shown as shaded boxes. Nucleotide numbering is based on the EcoRI restriction site as position 1.

The HBV genome contains three predicted CpG islands overlapping the start site of the S gene (island I); the region encompassing enhancer I, the X gene promoter (island II); and the region harboring the Sp1 promoter and the start codon of the P gene (island III) (**Figure 2**) (Vivekanandan et al., 2008b; Kaur et al., 2010). Kaur and his group in France reported a 14% methylation frequency in CpG island I, 0.6% methylation in island II and 3.7% in island III in CHB patients (Kaur et al., 2010). However, a computation study, reported that 50% of the HBV sequences examined lacked island I, while islands II and III were more conserved across genotypes (Zhang et al., 2013). The authors argued that conflicting results between HBV methylation studies are due to different HBV genotypes examined. High viral mutation frequencies and high viral replication rates in CHB infection can lead to a great degree of variability in CpG island distribution throughout the viral genome (Zhang et al., 2013).

The methylation status of island II has been greatly associated with reduced viral gene expression and replication as compared with islands I and III (**Table 1**). High methylation levels on island II were correlated with absent or low levels of HBsAg production while hypermethylation patterns were also reported in occult HBV cases and in HBsAg negative patients with HCC (Vivekanandan et al., 2008b; Kaur et al., 2010). In addition to transcriptionally regulatory genes, island II overlaps with the transcriptional control region of HBV cccDNA. Findings on the role of cccDNA methylation in HBV replication are controversial. Studies from several groups in Asia reported cccDNA methylation in human tissue and further showed that it impairs the cccDNA’s replication capability and viral gene expression (Guo et al., 2009; Vivekanandan et al., 2009; Kim et al., 2011). However, the study of Guo et al. (2009) included patients with a serum HBV DNA load of more than 10^3^ copies/ml, whilst Vivekandam and his group included liver cancer tissue; neither study made any correlation between cccDNA methylation and viral load, while both studies did not mention the presence of fibrosis. A third study from Kaur et al. (2010) included a small number of liver cirrhosis patients (*n* = 12), in which cccDNA methylation was associated with serum viremia. Contrary to these reports, a study from France, demonstrated that the HBV genome, including island II, is rarely targeted for DNA methylation in liver samples from CHB patients (Kaur et al., 2010). A Korean study of cirrhotic patients, reported that increased cccDNA methylation is associated with older age (Kim et al., 2011). The authors postulated that considering that perinatal transmission is the main mode of HBV infection and the stability of cccDNA, it is possible that methylated cccDNA may be passed to daughter cells after mitotic division, and additional methylation of replenished cccDNA may increase methylation frequency of the cccDNA population in chronic HBV infection (Kim et al., 2011). Furthermore, CpG island II is in close proximity to the core gene promoter and enhancer II and its hypermethylation can suppress Pre-C/C gene transcription, and consequently HBeAg expression in CHB patients (Guo et al., 2009). HBeAg seroconversion is attributed to hotspot mutations in the precore/BCP region (A1762T/G1764A, G1896A, G1899A) that abrogate or reduce HBeAg secretion (Jammeh et al., 2008). However, in the absence of these mutations cccDNA methylation density was reported to be significantly higher in liver cells of HBeAg(-) patients than in HBeAg(+) patients (Guo et al., 2009). Interestingly, within the same HBeAg(+) patient group, the ratio of RC-DNA/cccDNA was lower in the cccDNA methylation positive samples than in the cccDNA methylation negative samples (Guo et al., 2009). It is therefore possible that, increased cccDNA methylation correlates with HBeAg clearance possibly via suppressing pre-C/C transcription, which in turn can lead to suppressed viral productivity.

The specific roles of the DNMTs involved in the HBV methylation processes have not been clearly identified yet. Transfection studies reported that DNMT1, DNMT2 and DNMT3 expression is up-regulated in response to HBV, leading to viral methylation, decreased HBV gene expression, viral replication, and host DNA methylation (Vivekanandan et al., 2008a, 2010; Liu et al., 2009). Co-transfection experiments with HBV and DNMT3 were shown to increase cccDNA methylation and to down-regulation of viral protein and pgRNA production (Vivekanandan et al., 2010).

In addition, to stimulate transcription by modifying chromatin dynamics HBx protein has been shown to silence the expression of some genes by increasing the total DNMT activity of the host. Park et al. (2013) reported that HBx induced the upregulation of DNMT1, DNMT3A1 and DNMT3A2, and selectively facilitated the regional hypermethylation of the promoters of certain tumor suppressor genes through *de novo* methylation via DNMT3A1 and DNMT3A2 recruitment (Vivekanandan et al., 2010). In addition, Zheng and his group demonstrated that HBx regulated epigenetic modifications by its physical interaction with DNMT3A, either by promoting DNMT3A recruitment to the promoters of some genes, such as MT1F and IL-4, and thus inducing their hypermethylation and downregulation, or by preventing DNMT3A recruitment to specific genomic loci and thus activating the transcription of genes, such as CDH6 and IGFBP3 (Zheng et al., 2009). Fernadez and his group showed that most of the HBV genomes, although more methylated than the pre-malignant lesions, retained unmethylated the HBV gp3 gene, which codes for HBx (Fernandez et al., 2009). A recent study showed that the HBx protein impairs the expression of urokinase-type plasminogen (uPA), a serine protease essential for the activation of the hepatocyte growth factor (HGF) that activates hepatic regeneration (Park et al., 2013). HBx-induced uPA inhibition is regulated epigenetically by the hypermethylation of the uPA promoter (Park et al., 2013). In particular, in HBx-expressing cells, the CpG region of the uPA promoter was found to be 99.7% methylated resulting in the hypoactivation of pro-HGF and eventually hampering liver regeneration (Park et al., 2013).

## HBV Integration and Hepatocarcinogenesis

HCC is the third most common cause of cancer globally and chronic HBV patients have 100-fold greater risk of developing hepatocellular cancer. Ninety percept of HBV-associated liver cancers show integration of the HBV genome within the human genome (Fernandez et al., 2009). The development of HCC in HBV infection involves two major mechanisms: (1) the viral integration in the host’s genome causes *cis*-effects that inactivate tumor-suppressor genes and activate oncogenes and (2) the expression of trans-activating HBV proteins, such as the HBx and the PreS2 activators, which disrupt the signal transduction pathways and alter the expression of the infected hepatocyte (Schluter et al., 1994; Park et al., 2013).

Similarly to other oncoviruses (HPV16 and HPV18), the HBV genome, is almost unmethylated in the early stages of carcinogenesis while it becomes more methylated in the established HCC (Fernandez et al., 2009). Hepatitis C virus (HCV) contributes to carcinogenesis by inducing regional hypermethylation of CpG islands in the promoter regions of multiple genes (Calvisi et al., 2007). In HBV-induced carcinogenesis, HBx can accelerate hepatocarcinogenesis epigenetically by promoting hypermethylation of tumor suppressor genes by modulating DNMT1 and DNMT3A expression (Park et al., 2013). Moreover, HBx was reported to induce hypermethylation of the E-cathedrin promoter via the activation of DNMT1 *in vitro* (Lee et al., 2005). In addition to DNMT expression, high HBx expression has been correlated with the hypermethylation of the promoter of the major tumor suppressor gene p16^INK4A^ and the subsequent reduction in p16 protein expression in non-cancerous tissue but not in HCC tissue suggesting that HBx plays an important role in the early stages of HBV associated HCC (Zhu et al., 2007).

HCC has been associated with high methylation of CpG island I which overlaps with the HBsAg gene starting site (Kaur et al., 2010). Another study, confirmed the progressive presence of hypermethylation at the HBV gp2 locus that encodes for S viral proteins in primary liver tumors (Fernandez et al., 2009).

## HBV Epigenetic Control of Host’s Immune Responses

Host DNMT upregulation by viruses can be a non-specific innate response to infection. HBV has been shown to induce genome-wide DNA methylation changes, including immunoregulatory genes that are active against HBV (Ancey et al., 2015). In particular, HBV replication was reported to cause the *de novo* methylation and decrease of IL4, which benefits the virus since IL4 expression inhibits HBV replication (Zheng et al., 2009). In addition, unmethylated CpG dinucleotides have been shown to trigger toll-like receptors expressed in hepatic cells *in vitro*, which in turn can activate the NF-κB pathway that plays a key role in the innate system’s ability to inhibit HBV replication (Liaw et al., 2009). Nevertheless, this potentially protective effect of DNMT upregulation may be offset over time either through viral manipulation of the host methylation machinery or through non-specific methylation of host CpG islands as a result of chronic over expression of DNMTs. For example, latent viruses, such as Epstein-Barr virus (EBV), are maintained in their latent state in part by methylation, suggesting that some viruses have evolved strategies to manipulate host DNMTs to their advantage. HBV viral proteins can lead to DNMT upregulation and eventually to methylation of the host genes, including oncogenes. A recent study by Tropberger and his group showed that transcription and active PTMs in cccDNA are reduced by the activation of an innate pathway, and that this effect can be recapitulated with a small molecule epigenetic modifying agent (Tropberger et al., 2015).

## Therapeutic Implications of Epigenetic Mechanisms in HBV Infection

The most important goal in HBV research is the development of therapies to eradicate HBV infection. Considering the long half-life of the hepatocytes, the limiting factor in eliminating infection is the clearance of the cccDNA pool from the infected cells. Therefore, interfering with the epigenetic regulation of the cccDNA minichromosome is the most promising therapeutic approach. Experiments in humanized mice and cell culture demonstrated that treatment with IFN-α induces cccDNA-bound histone hypoacetylation and the active recruitment of transcriptional co-repressors onto cccDNA (Belloni et al., 2012). IFN-α administration was also shown to reduce binding of STAT1 and STAT2 transcription factors to active cccDNA (Chen et al., 2013). Treatment with IFN and other potential cytokines that can activate the cellular response via the epigenetic modifications of cccDNA could mark the episome for selective eradication of infected cells or prevent cccDNA molecules from re-entering into nuclei after mitosis. Epigenetic alterations could also potentially alter cccDNA partitioning into daughter cells. Exploring the molecular mechanisms by which IFN-α mediates epigenetic repression of cccDNA transcriptional activity and identifying new molecular determinants can lead to the development of a treatment that would eradicate cccDNA molecules.

In CHB infection, viral and host DNA is a host defence mechanism to suppress viral replication. Increased expression of DNMTs has been reported in CHB livers that facilitates viral genome methylation and affects protein production and viral replication (Guo et al., 2009; Vivekanandan et al., 2010). Furthermore, host DNA methylation has been shown to be the main mechanism inactivating relevant genes in HCC, suggesting a potential role of strong demethylating agents in the treatment of HCC (Claus et al., 2005; Tong et al., 2009). However, such potent demethylating treatment could lead to the reactivation of HBV replication. Confirming that methylation of non-integrated HBV genomes can regulate viral replication and cccDNA transcription leads to several significant clinical correlates. Dietary consideration may potentially be important in modulating HBV replication, as dietary deficiencies can limit the liver’s ability to methylate HBV. Mouse experiments showed that dietary supplementation with folate, vitamin B12, choline and betaine could lead to host gene methylation (Waterland, 2006). These findings indicate a potential role of methylation in the future treatment of CHB infection.

## Concluding Remarks

In the last decade our knowledge on epigenetic modifications in viral infection has increased dramatically. DNA methylation and histone modifications have been shown to play important roles in regulating the expression of a variety of HBV genes and viral replication. The holy grail of the future of HBV therapy is the complete elimination of cccDNA of all the infected cells in the host. The association of the cccDNA acetylation changes with viral replication and transcription shows that the dynamic acetylation and deacetylation of cccDNA-associated histones is essential in cccDNA transcription. Additionally, IFN-α has been shown to actively decay cccDNA due to acetylation modifications but the exact mechanisms and the role of cell division in cytokine-induced cccDNA elimination remain to be determined. DNA methylation is also being increasingly recognized to play a role in regulation of HBV gene expression. Both integrated and episomal HBV DNA can be methylated in human tissues and high HBV DNA methylation associates with HCC development. Studies on cccDNA methylation have provided conflicting results mainly because the content of cccDNA in the infected hepatocytes is maintained by the *de novo* cccDNA synthesis and possibly due to the different HBV genotypes and experimental approaches applied. HBx has been shown to play a key role in the acetylation and methylation of cccDNA either directly by being recruited onto the cccDNA minichromosome and indirectly by the modulation of epigenetic-associated proteins, including DNMTs and cccDNA-bound histones. Enhancing our understanding of the epigenetic consequences of HBV-host interactions will lead to the identification of novel potential therapeutic targets for selective inhibition of cccDNA transcription and therefore complete eradication of HBV infection. Since the epigenetic processes are reversible they would also provide new molecular determinants by which host and environmental factors can regulate HBV replication and pathogenesis.

## Conflict of Interest Statement

The authors declare that the research was conducted in the absence of any commercial or financial relationships that could be construed as a potential conflict of interest.
